# Postoperative Outcomes of a Digital Rehabilitation Program After Total Knee Arthroplasty: Retrospective, Observational Feasibility Study

**DOI:** 10.2196/40703

**Published:** 2022-09-19

**Authors:** Mindy Hong, Joey Loeb, Manshu Yang, Jeannie F Bailey

**Affiliations:** 1 Hinge Health, Inc San Francisco, CA United States; 2 Department of Psychology University of Rhode Island Kingston, RI United States; 3 Department of Orthopaedic Surgery University of California San Francisco San Francisco, CA United States

**Keywords:** total knee arthroplasty, surgical, digital intervention, musculoskeletal, telemedicine

## Abstract

**Background:**

Surgery can sometimes be the best solution for chronic musculoskeletal pain, but presurgical preparation and postsurgical rehabilitation are often required to achieve the maximum benefits. A digital musculoskeletal surgical care program was developed to support the population of patients undergoing total knee arthroplasty.

**Objective:**

We aimed to demonstrate safety, engagement, and acceptability and explore clinical outcomes, health care use, and satisfaction among participants of a digital musculoskeletal surgical care program who were undergoing total knee arthroplasty.

**Methods:**

A retrospective, observational feasibility study comparing digital musculoskeletal surgical care program participants to a comparison group was conducted. The intervention group registered for a digital musculoskeletal surgical care program, which included health coaches, physical therapists, and tailored exercises and educational articles to provide preoperative and postoperative support to patients who had recently undergone total knee arthroplasty. Comparison group members received standard-of-care treatment. Engagement (number of exercise therapy sessions and educational articles accessed per week) and acceptability (Net Promoter Score) were examined among intervention group participants. Descriptive statistics for postoperative outcomes, including safety (postoperative complications), clinical improvement (pain, function, anxiety, and depression), and health care use and experiences (length of hospital stay, surgery satisfaction, and physical therapy adherence), were reported for both groups. Differences among postoperative results were compared by using the independent samples 2-tailed *t* test or Mann-Whitney test for continuous outcomes and the Fisher exact test or chi-square test for categorical outcomes.

**Results:**

Of the 53 participants (intervention group: n=22; comparison group: n=31) who were included in this study, 35 (66%) were female and 25 (47%) were aged from 45 to 60 years. On average, the intervention group completed 23 exercise sessions, read 2.7 educational articles, sent 45.5 texts to their health coaches, and were actively engaged for 6 weeks after their operation. Among 21 participants, 14 (67%) self-reported as *promoters* on the Net Promoter Score scale. Intervention group members reported fewer postoperative complications (6/22, 27%) than the comparison group (15/31, 48%), and they experienced better outcomes with regard to function (Knee Injury and Osteoarthritis Outcome Score–Physical Function Short Form—intervention group: mean 23.0; comparison group: mean 32.5), depression (Patient Health Questionnaire 2-Item—intervention group: mean 0.4; comparison group: mean 1.6), anxiety (General Anxiety Disorder 2-Item—intervention group: mean 0.6; comparison group: mean 1.5), and impressions of change (Patient Global Impression of Change—intervention group: median 7.0; comparison group: median 6.0). Intervention group participants also reported less health care use, better adherence to their physical therapy exercises, and higher surgery satisfaction.

**Conclusions:**

Our digital musculoskeletal surgical care program shows promising levels of engagement and acceptability among those who recently underwent total knee arthroplasty. The surgical care program may also help with improving postsurgical complications and clinical outcomes and lowering health care use.

## Introduction

Chronic musculoskeletal pain is a leading cause of disability and increased health care costs in the United States, affecting over 50 million people and resulting in an estimated total lost productivity cost of US $296 million per year [1]. Evidence-based clinical guidelines typically recommend performing nonsurgical interventions before performing invasive procedures for people with chronic musculoskeletal pain [2]. However, these treatments are not always effective for everyone, and when these treatments are inadequate, surgery can be recommended [3]. For example, total knee arthroplasties have proven to be successful in improving pain, mobility, and quality of life for many with chronic knee pain [4,5]. However, despite substantial improvements in surgical techniques and treatments, approximately 20% of patients report dissatisfaction following total knee arthroplasty [5,6]. The causes of dissatisfaction appear to be multifactorial; unmet expectations, the inability to engage in postoperative rehabilitation, and limited pain relief can affect satisfaction after total knee arthroplasty [6,7].

For surgery outcomes to be successful, it is important for patients to adhere to both preoperative rehabilitation and postoperative rehabilitation [5,8-11]. However, almost half of adults do not engage in and adhere to postoperative rehabilitation [12]. This is due to gaps in social support, poorly managed expectations, and a lack of education regarding the benefits of postoperative rehabilitation [7,13]. Furthermore, despite the benefits of receiving social support after total knee arthroplasty [14], not all patients have the social support they need.

**Figure 1:**
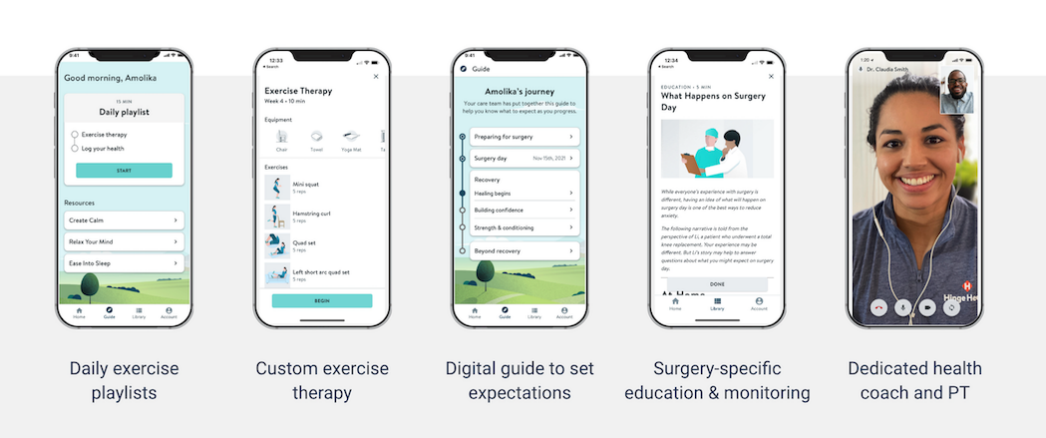
The digital musculoskeletal surgical care program provides users with a tailored program containing medical, social, and educational support. PT: physical therapist.

In order to address care gaps and their subsequent impacts on both care quality and clinical outcomes, we developed a digital surgical care program consisting of medical, social, and educational support (Figure 1). The digital musculoskeletal surgical care program’s goal was to support and help patients throughout the preoperative and postoperative stages of surgery. The program included surgical health coaches and certified physical therapists who worked with participants to help them prepare for and recover from surgery, answer questions, and customize participants’ plans of care. In addition, the program also included customized exercise therapy sessions that followed the protocols created by participants’ surgeons and aimed to strengthen and rehabilitate participants. The participants also received tailored education articles on lifestyle management and recovery tips that helped prepare them for the preoperative and postoperative phases of surgery. All materials and services were accessed through the digital musculoskeletal surgical care program app.

The preoperative phase of the surgical care program started up to 8 weeks prior to surgery. Participants worked with health coaches to achieve goals that supported the best surgical outcomes, such as reducing presurgery anxiety or creating healthier eating habits for recovery. Participants also worked with physical therapists to strengthen muscles that supported their new joints to allow for optimal recovery. The postoperative phase lasted for 12 weeks after surgery. During this time, participants received support and accountability training for achieving their recovery goals, as per their rehabilitation plans.

The program was complemented by the plans that were recommended and directed by participants’ surgeons. As such, participants still attended their recommended in-person physical therapy sessions. Participants informed their care program health coaches of their in-person physical therapy exercises, and information on these exercises was loaded into the app to assist with tracking progress.

The goal of this study was to determine the feasibility of our novel surgical care program. The primary objective was to demonstrate safety, engagement, and acceptability among the participants of the surgical care program. The secondary objectives included exploring postoperative clinical improvement and comparing the health care use and experiences of surgical care program participants against those of a comparison group.

Patients who need orthopedic surgery typically benefit from the social support provided through caregivers or family members, but not everyone is able to receive assistance. Therefore, our digital surgical care program aims to provide the needed social support and improve care quality and clinical outcomes both before and after the surgery process. The results from this study will contribute to developing larger-scale studies that will provide evidence that this digital surgical care program effectively addresses care gaps.

## Methods

### Study Design

A retrospective, observational feasibility study comparing digital musculoskeletal surgical care program participants (herein, the *intervention group*) to a comparison group was conducted.

### Study Participants

Participants who were eligible for the intervention were identified based on our inclusion and exclusion criteria and the information they provided in the app. The inclusion criteria were patients who had a smartphone, patients who created an account for the digital musculoskeletal surgical care program, patients who completed the app questionnaire at least 4 weeks prior to starting the surgical care program, patients who enrolled in the knee surgical care program, patients who underwent knee replacement surgery 6 or more weeks before survey data collection, and English-speaking patients.

Comparison group participants were recruited through a proprietary research panel maintained by Momentive. The panel includes over 100 million people who are invited to take part in surveys. We screened panelists by age and included those who had not previously participated in Hinge Health programs and underwent knee replacement surgery between June and December 2021.

### Ethics Approval

This study (reference number: 20160949) was reviewed and approved by the WIRB-Copernicus Group Institutional Review Board (Office for Human Research Protections and Food and Drug Administration Institutional Review Board registration number: IRB00000533). Intervention group participants acknowledged and provided research consent. The institutional review board deemed the comparison group participants exempt from providing informed consent.

### Variables

#### Overview of Outcomes

For the primary objective of this feasibility study, we examined engagement, acceptability, and safety outcomes to demonstrate that it was possible to implement the program and this study*.* We also explored proximal and distal outcomes that we hypothesized would be influenced by the program. These included clinical outcomes (pain, function, depression, and anxiety), health care use (length of hospital stay and physical therapy adherence), and health care experiences (surgery satisfaction). This study collected data on outcomes regarding engagement and acceptability from the intervention group. Data on safety, clinical improvement, and health care use and experience outcomes were collected from both the intervention group and the comparison group.

#### Engagement and Acceptability

To assess the intervention group’s engagement, data on the number of app-based exercise therapy sessions and educational articles accessed per week were collected through the app. Acceptability was measured through the Net Promoter Scores (NPSs) that participants provided for the following question: “On a scale of 0-10, how likely is it that you would recommend the surgical program to a friend or colleague” (0=not likely; 10=very likely)? NPSs ranging from 0 to 6 were labeled as *detractors*, NPSs of 7 to 8 were labeled as *passives*, and NPSs of 9 to 10 were labeled as *promoters*.

#### Safety

Safety was based on self-reported postsurgical complications, which included wound infections requiring antibiotics or surgery, blood clots or deep vein thrombosis, stiffness requiring manipulation under anesthesia, falls, surgery on the same knee for other reasons, and general soreness.

#### Clinical Improvement

The clinical outcomes included pain improvement, function, anxiety, and depression. Patient Global Impression of Change (PGIC) scores were measured for both groups through the following question: “Compared to before the surgery, how would you rate your knee pain now” (1=much worse; 7=much improved)? Function was measured through the Knee Injury and Osteoarthritis Outcome Score–Physical Function Short Form (KOOS-PS; 0=no difficulty; 100=extreme difficulty). Anxiety and depression were measured through the General Anxiety Disorder 2-Item (GAD-2) and Patient Health Questionnaire 2-Item (PHQ-2), respectively.

#### Health Care Use and Experiences

We measured the length of stay in the hospital after surgery based on the following question: “How many nights did you spend in the hospital or surgery facility after your surgery?” Surgery satisfaction was measured based on the following question: “Overall, how satisfied were you with the results of your knee replacement surgery” (0=very dissatisfied; 3=very satisfied)? Physical therapy adherence was measured through the following question: “How often did you perform home exercise sessions as recommended by your in-person physical therapist” (0=never; 5=always)?

### Data Sources

The web-based program registration process provided baseline demographic data for the intervention group. Surveys that evaluated postoperative outcomes were emailed to both the intervention group and the comparison group. Intervention group respondents received gift cards worth US $30 upon the completion of the survey.

### Statistical Methods

Because this was a feasibility study, no formal sample size calculations were conducted. Summary statistics were estimated for the baseline demographic characteristics of the intervention group. Descriptive statistics for the postoperative outcomes of both the intervention group and the comparison group were reported, including means with SDs and medians with IQRs. Differences between the postoperative results of the two groups were compared by using the independent samples 2-tailed *t* test or Mann-Whitney *U* test for continuous or ordinal outcomes. Categorical variables were analyzed by using the Fisher exact test or chi-square test. Analyses were performed in R version 4.0.5 (R Foundation for Statistical Computing).

## Results

### Sample Characteristics

Altogether, 53 participants completed the study (intervention group: n=22; comparison group: n=31). Table 1 shows the demographics and the months of surgery for both groups. Apart from age (intervention group participants were significantly older than comparison group participants; *P*<.001), no differences between the two groups were detected at baseline. The majority of the sample was aged from 45 to 60 years (25/53, 47%), was female (35/53, 66%), and received surgery in September (11/53, 21%) and October (11/53, 21%).

**Table 1:** Study participant characteristics.

Characteristics			Intervention group (n=22), n (%)		Comparison group (n=31), n (%)		All participants (N=53), n (%)		*P* value	
**Age (years)**										<.001^a^
	30-44	0 (0)		4 (13)		4 (8)				
	45-60	5 (23)		20 (65)		25 (47)				
	>60	17 (77)		7 (23)		24 (45)				
**Sex**										.57^b^
	Male	6 (27)		12 (39)		18 (34)				
	Female	16 (73)		19 (61)		35 (66)				
**Month of surgery^c^**										.99^a^
	June	1 (5)		2 (7)		3 (6)				
	July	3 (14)		5 (16)		8 (15)				
	August	2 (9)		4 (13)		6 (11)				
	September	4 (18)		7 (23)		11 (21)				
	October	5 (23)		6 (19)		11 (21)				
	November	4 (18)		3 (10)		7 (13)				
	December	3 (14)		4 (13)		7 (13)				

^a^Fisher exact test.

^b^Chi-square test with continuity correction.

^c^Months of surgery are from 2021.

### Engagement and Acceptability (Intervention Only)

On average, intervention group participants completed 23 (SD 34.8) exercise therapy sessions, read 2.7 (SD 5.3) educational articles, sent 45.5 (SD 51.7) text messages to their health coaches, and were actively engaged for 6 (SD 6.7) weeks after their operation. Among 21 program participants, 14 (67%) were promoters on the NPS.

### Safety

The percentage of participants who reported postsurgical complications was higher in the comparison group versus the intervention group by 21%. The reported complications among comparison group members were stiffness (4/15, 27%), surgery on the same knee for other reasons (4/15, 27%), blood clots or deep vein thrombosis (4/15, 27%), wound infections requiring antibiotics (2/15, 13%), general soreness (2/15, 13%), infections requiring surgery (1/15, 7%), and falls (1/15, 7%). The complications among the intervention group were stiffness (4/6, 67%) and vasculitis (ie, a latex allergy; 1/6, 17%). Manipulation for addressing stiffness was recommended for a patient, but the treatment was deferred (1/6, 17%).

### Clinical Improvement

The intervention group reported better knee pain after their surgery compared to that reported by the comparison group (PGIC score: mean 7.0 vs mean 6.0; *P*=.06). The intervention group reported better function scores (KOOS-PS: mean 23.0 vs mean 32.5; *P*=.049) than those reported by the comparison group. Intervention group members also reported lower anxiety (GAD-2 score: mean 0.6 vs mean 1.5; *P*=.01) and depression (PHQ-2 score: mean 0.4 vs mean 1.6; *P*=.004) scores than those reported by the comparison group.

### Health Care Use and Experiences

The median length of hospital stay was 1 (IQR 0) night for the intervention group and 1 (IQR 1) night for the comparison group. There was a significant difference (W=275; *P*=.009) in the lengths of hospital stay between the intervention group and the comparison group. Additionally, the intervention group showed better adherence to the exercises that were recommended by their physical therapists (*P*=.06) and reported higher satisfaction with their surgery experience (*P*=.06). Table 2 shows the postoperative outcomes of the intervention and comparison groups.

**Table 2:** Postoperative outcomes.

Outcomes		Intervention group (n=22)	Comparison group (n=31)	All participants (N=53)	*P* value	
**Safety (postsurgical complications), n (%)**						.12^a^
	No complications	16 (73)	16 (52)	32 (60)		
	Complications	6 (27)	15 (48)	21 (40)		
**Clinical outcomes**						
	Patient Global Impression of Change score, median (IQR)	7.0 (1.0)	6.0 (2.0)	7.0 (1.0)	.06^b^	
	Knee Injury and Osteoarthritis Outcome Score–Physical Function Short Form, mean (SD)	23.0 (17.4)	32.5 (15.9)	28.6 (17.0)	.049^c^	
	General Anxiety Disorder 2-Item score, mean (SD)	0.6 (1.3)	1.5 (1.6)	1.1 (1.5)	.01^b^	
	Patient Health Questionnaire 2-Item score, mean (SD)	0.4 (1.1)	1.6 (1.8)	1.1 (1.7)	.004^b^	
**Health care use and experiences**						
	Length of hospital stay (number of nights), median (IQR)	1.0 (0)	1.0 (1.0)	1.0 (0)	.009^b^	
	Physical therapy adherence score, median (IQR)	4.0 (2.0)	3.0 (2.5)	3.0 (3.0)	.06^b^	
	Surgery satisfaction score, median (IQR)	3.0 (1.0)	2.0 (1.0)	2.0 (1.0)	.06^b^	

^a^Chi-square test.

^b^Mann-Whitney *U* test.

^c^Independent samples *t* test.

## Discussion

### Principal Results

This study aimed to assess the feasibility of a novel, digital surgical care program for total knee arthroplasty by (1) examining patient engagement, acceptability, and safety and (2) exploring clinical improvements as well as health care use and experiences. First, we posit that a program that offers clinical, educational, and social support is viable and that it would be well received by persons planning to undergo a total knee arthroplasty. We found that on average, the intervention group remained active in the program by engaging in 23 exercise sessions, reading 2.7 educational articles, and sending 45.5 text messages to their coaches. Satisfaction among the intervention group was high, as two-thirds of participants (14/21, 67%) were NPS promoters.

We also demonstrated that the intervention was safe. The most frequently reported complication from the intervention group was stiffness, which can be expected postoperatively. Manipulation under anesthesia for stiffness was recommended for a patient by the surgeon but was deferred by the patient. The remaining complication (a latex allergy) was unrelated to the surgery itself. The intervention group also experienced fewer adverse events and complications than those experienced by the comparison group. One possible explanation is that the intervention helped participants adhere to postsurgical exercise regimens and avoid complications. Another explanation is the presence of unmeasured confounding variables. For example, intervention group members may have received a higher quality of in-person care compared to the care that the comparison group received.

The results of this study will be used in two formative projects. In the first project, we will use the lessons learned in this study to refine and improve the program. For example, we learned more about the nature of the complications experienced by both the intervention group and the comparison group. We may prepare additional tools and resources for participants about how to identify complications, when to contact providers for help, and how to best recover from complications. In the second project, we will use the estimates from this preliminary study to develop a larger observational study that is powered to detect statistically significant differences between an intervention group and comparison group. Furthermore, implementing the surgical care program at scale will allow us to further evaluate intervention effectiveness in a more generalizable setting.

### Comparison With Prior Work

We provide preliminary evidence that an intervention that offers clinical and social support is associated with better clinical outcomes, including better impressions of change, function, anxiety, and depression scores after surgery. These results are consistent with those of a qualitative study that found that patients who had a positive total knee arthroplasty experience often reported having adequate social and clinical support [15]. These results are also consistent with those of studies reporting that social support has a positive impact on pain, function, mental health, and patient satisfaction among patients who have undergone joint replacement surgery [14-16]. A meta-analysis found that the presence of social support had a beneficial effect on total Western Ontario and McMaster Universities Arthritis Index scores (mean difference: 2.88, 95% CI 1.30-4.46), which are used to measure pain, functional limitations, and stiffness. The same meta-analysis also found social support to be positively associated with improvements in knee pain and function (total Oxford Knee Scores—mean difference: 0.29, 95% CI 0.12-0.45) [14]. We speculate that improvements in clinical outcomes could be the result of having a care team that consists of health coaches, physical therapists, and physicians who support patients and offer medical advice throughout recovery. Our study also explored health care use and experiences and showed shorter lengths of hospital stay, better physical therapy adherence, and higher levels of satisfaction in the intervention group. Similarly, studies have reported that inadequate social support and unmet expectations result in higher rates of dissatisfaction, poorer adherence to outpatient therapy, and poorer outcomes [5-7,10]. In a prospective study of 1703 participants that examined satisfaction after total knee arthroplasty, unmet expectations was one of the strongest predictors of dissatisfaction in a comparison between dissatisfied and satisfied participants (49% vs 6%) [5].

### Strengths and Limitations

The strengths of this study include evaluating multiple outcomes to demonstrate program feasibility, including engagement, acceptability, safety, clinical, and health care use and experience outcomes. This study also included a comparison group for comparing postoperative outcomes.

This study however also presents limitations. As with most retrospective studies, there is a risk of bias associated with data that are collected prior to the start of a study. This study compared the postoperative outcomes of the intervention and comparison groups. A future, larger-scale observational study that compares both preoperative outcomes and postoperative outcomes with those of a comparison group, as well as surgeries in other pathways (eg, total hip arthroplasty), can provide more insight into the effectiveness of a digital musculoskeletal surgical care program. Lastly, this study was designed to demonstrate feasibility with a small set of early surgical care program members and a convenience sample comparison group. As such, there are differences between the two groups that may have influenced our exploratory clinical outcomes. The preliminary findings of this study are encouraging, and they will be used as the basis for the next steps in program development, that is, in research where we will adjust for potential confounding factors.

### Conclusions

Our digital musculoskeletal surgical care program, which provides social support, medical advice, and education to those who have recently undergone total knee arthroplasty, is feasible and acceptable. We demonstrated engagement, satisfaction, and safety among the participants of the program. Additionally, compared to the comparison group, this study showed preliminary evidence of improved clinical outcomes, lower health care use, and higher satisfaction among intervention group participants. Based on the results of this feasibility study, a larger-scale observational study can build on the findings of this study to further evaluate the effectiveness of the surgical care program.

## Abbreviations

GAD-2: General Anxiety Disorder 2-Item
KOOS-PS: Knee Injury and Osteoarthritis Outcome Score–Physical Function Short Form
NPS: Net Promoter Score
PGIC: Patient Global Impression of Change
PHQ-2: Patient Health Questionnaire 2-Item
